# δ^13^C and δ^15^N Values of Residues Provide Insights Into Identification of the Explosive Source

**DOI:** 10.1002/rcm.70057

**Published:** 2026-02-25

**Authors:** James R. Ehleringer, John D. Howa

**Affiliations:** ^1^ School of Biological Sciences, University of Utah Salt Lake City Utah USA; ^2^ Sports Medicine Research and Testing Laboratory South Jordan Utah USA

**Keywords:** HMX, PETN, RDX, stable isotopes, TNT

## Abstract

**Rationale:**

Postblast analyses of military and terrorist events will benefit from the capacity to learn more about the explosive materials used in an event. Here stable isotope ratio analyses (δ^13^C, δ^15^N) can provide additional information to complement identification of the explosive components.

**Methods:**

Controlled detonations using different types of military‐grade explosives were conducted in 55‐gal barrels. Additionally, soil analyses were conducted following Mark‐84 field detonations. Swab materials and soils were purified to analyze explosive compounds using established HPLC and IRMS techniques.

**Results:**

Explosive materials were recovered in the residues of TNT (aromatic‐based explosive) and RDX (nonaromatic explosive) detonations in barrel experiments. Explosive residues were not recovered from PETN (nonaromatic explosive). Postblast δ^13^C and δ^15^N values of TNT residues were similar to δ values in the source explosive, suggesting minimal enrichment in δ residues. While δ^15^N values of RDX in postblast residues were also similar to preblast source values, postblast RDX δ^13^C values were enriched by almost 2‰ relative to the preblast explosive. Similar patterns were observed in HMX, RDX, and NT recovered from soils following Mark‐84 detonations.

**Conclusions:**

δ^13^C and δ^15^N values can be effectively measured on explosive compounds recovered in residues following detonations. Residue δ^13^C and δ^15^N values can be linked to δ values of undetonated explosive compounds. Additional field studies should be conducted to verify these results.

## 1 Introduction

Stable isotope ratio analyses have been frequently applied to forensic investigations [1, 2, 3, 4, 5, 6, 7]. Among those applications are explorations of the variations in stable isotope ratios of explosive compounds. These span a range of explosive materials associated with bombings and IEDs, including TNT [8, 9, 10], commercial and military grade (HMX, PETN, RDX) [10, 11, 12, 13], ammonium nitrate (AN) [14, 15, 16, 17, 18], and triacetone triperoxide (TATP) [19, 20, 21, 22] explosives.

Stable isotope analyses of bulk explosives alone are likely to provide limited forensic information without compound‐specific analyses and reference to a database. This is because explosives are often mixtures of explosive compounds, including stabilizers, waxes, stearates, and phthalates, each of which is likely to have different stable isotope compositions. Chesson et al. [23] and Howa et al. [24] provide a solution describing a framework for separating compounds within explosive materials so that (a) HPLC methods could identify and quantify each of the components in the mixture and (b) IRMS analyses could provide stable isotope ratios of explosive components, effectively creating a fingerprint. This approach has been applied to the analyses of military grade and IED explosives.

At the same time, initial postblast investigations have been conducted using well accepted approaches to sample explosive residues and to identify the primary explosive compounds involved in the detonation [25, 26, 27]. The first known publication exploring preblast and postblast residues of explosives common in ordnance was by McGuire et al. [28]. While that study compared postblast residues following detonations with explosives containing HMX, TATB, and TNT, the postblast material was soot, containing both black carbon and any of the remaining explosive compounds. Thus, the conclusions were tentative. More recently, Oxley et al. [29] explored postblast stable isotope ratios of both DNAN and TNT recovered from residues in separate experiments. Their exciting results revealed that recovered postblast residues of both DNAN and TNT had stable isotope ratios similar to the preblast explosive compounds. Their results indicate the potential for stable isotope ratio analyses in postblast investigations to be a valuable analytical measure in helping to trace the explosive or IED back to specific sources. Similarly, stable isotope analyses of AN revealed that pre‐ and purified postblast samples were of similar isotopic composition [30, 31].

This study was designed to address three basic questions related to postblast analyses: (Question 1) Is sufficient material recoverable from residues following a detonation to determine the stable isotope ratios of explosive compounds? (Question 2) If explosive compounds can be recovered and measured, how do preblast and postblast stable isotope ratios compare? (Question 3) Can postblast stable isotope ratio observations be related back to their manufacturer? Our guiding hypothesis is “Following a detonation, undetonated explosive materials can be recovered from residues and isotopically measured with high precision.”

## 2 Methods

### 2.1 Explosives Database

An explosive database was constructed using small amounts (< 5 g) of HMX and RDX obtained (a) through visits to production facilities in Switzerland, United Kingdom, and USA and (b) via samples acquired from the US Federal Bureau of Investigation, US Bureau of Alcohol, Tobacco, and Firearms, and the US Department of Defense. Authority to receive and store explosive samples was granted through ATF license 9‐UT‐035‐33‐0L‐00349. Since completion of this study, the database and authentic explosive materials have been relinquished to the US Government.

Purification, identification, and stable isotope analyses of all explosives presented in this publication were conducted following protocols described by Chesson et al. [23] and Howa et al. [24].

### 2.2 Controlled Detonations in Barrels

Controlled detonations were conducted in new 55‐gal barrels. In each experiment, explosive materials were wrapped around a detonator and suspended approximately one‐third from the height of an inverted barrel. A total of 50 experiments were conducted, each with a new 55‐gal barrel. Approximately 100 kg of metal was placed on the inverted barrel so that following the detonation, the barrel only flew 4–8 m into the air, making recovery of the residue‐containing barrel feasible. The mass of the explosives used in each experiment was 5, 10, or 20 g.

Twelve different military grade explosives were provided for these experiments by the US government. These included aromatic‐based explosives: (a) RDX‐containing explosives—C3, C4, Flex‐X M186, military dynamite, and Primasheet RDX and (b) PETN‐containing explosives—Detcord, Semtex 10, Semtex A, Semtex H, Flex‐X M118, and Primasheet PETN; and nonaromatic explosives: (c) TNT‐containing explosives—military TNT and TNT flaked.

Cotton balls were used to collect residues following a detonation experiment. Cotton balls to be used for residue collections were first soaked in deionized water for 2 h. The cotton balls were then dried by pressing them into a Büchner funnel attached to a flask under vacuum. The cotton balls were then soaked in acetonitrile for 2 h. The acetonitrile was then decanted away by again pressing them into a Büchner funnel attached to a flask under vacuum. Lastly, the cotton balls were dried in an oven at 50°C for 18–20 h. Shortly after conducting the field experiments, the cotton balls were pretreated with 0.5–0.7 mL of an 80:20 isopropanol:water mix. The cotton swabs were then stored in 10‐mL plastic syringes, capped at each end.

Following each detonation, the insides of the barrels were sampled for residues. The insides of each barrel were divided into six sections, with each section swabbed using a single water‐soaked cotton ball. One swab was used for each of these portions of the interior of the barrel: top, bottom (lid), upper one‐third, top middle‐sixth, bottom middle‐sixth, and lower one‐third. Residue‐containing swabs were each placed in 10‐mL plastic syringes and capped at each end. These samples were then stored in a cool environment until residue extraction could occur back at our University of Utah laboratory. In a separate experiment, new barrels were randomly swabbed for explosives; none were detected.

### 2.3 Collection and Storage of Soil Residues Following Mark‐84 Detonations

Five Mark‐84 bombs, each weighing 2000 pounds, were detonated at a separate location within a US Government facility. Following each detonation, 1 kg of topsoil from the rims of craters was collected and sent to our laboratory at the University of Utah. The soils were dried at 70°C and 250 g of soil was used for HPLC and IRMS analyses.

### 2.4 Extraction and Analyses of Explosive Residues

In the laboratory, the residue‐containing swabs were extracted following the US FBI protocol described by Thompson et al. [27]. Each swab‐containing syringe was purged with 20 mL of deionized water. The water was collected and then centrifuged to precipitate the solid residue materials. The supernatant was injected into a solid phase extraction syringe with the fluid then purified using HPLC and compound‐specific stable isotope ratio analyses.

#### 2.4.1 Sample Purification

Liquid samples containing explosive compounds were identified, quantified, and their stable isotope ratios using protocols described by Chesson et al. [23] and Howa et al. [24].

Soil samples were also analyzed using the same extraction procedures, which allowed for determinations of cyclohexane‐soluble, acetone‐soluble, and water‐soluble explosive compounds.

#### 2.4.2 Stable Isotope Analysis

Carbon and nitrogen isotope ratio analysis was measured concurrently at the Stable Isotope Ratio Facility for Environmental Research (SIRFER) at the University of Utah on a Thermo Finnigan Delta S isotope ratio mass spectrometer (Bremen, Germany) attached via ConFlo interface to an EA with an autosampler (Carlo Erba, Milan, Italy). EA reactors were packed according to the manufacturer's specifications with chromium oxide and silvered cobaltous/cobaltic oxide in the oxidation reactor and reduced copper wire in the reduction reactor. Magnesium perchlorate removed water upstream from a 3‐m ¼″ packed GC column used to separate N_2_ and CO_2_ gases. Stable isotope ratios are expressed in “delta” (δ) notation in ‰ (per mil) relative to a standard, where *δ* = (*R*_A_ ‐*R*_Std_)/*R*_Std_ and *R*_A_ and *R*_Std_ are the ratios of the rare to abundant isotope (e.g., ^13^C/^12^C or ^15^N/^14^N) in the sample and the standard, respectively. The international standard used for carbon is Vienna Pee Dee Belemnite (VPDB) and the standard for nitrogen is atmospheric N_2_ (Air). Samples were analyzed alongside an imidazole reference material, which was used to normalize sample δ^13^C and δ^15^N data to international isotope scales. We also included a pure PETN reference material to monitor instrument stability and estimate long‐term analytical uncertainty (given below). We used a two‐point normalization for δ^13^C and δ^15^N data using the glutamic acid reference materials USGS40 and USGS41 (available from nist.gov as SRM8573 and SRM8574). Measured isotope ratios for imidazole and previously analyzed samples were corrected using these reference materials.

## 3 Results and Discussion

### 3.1 Isotope Variations Among Production Sources

**FIGURE 1:**
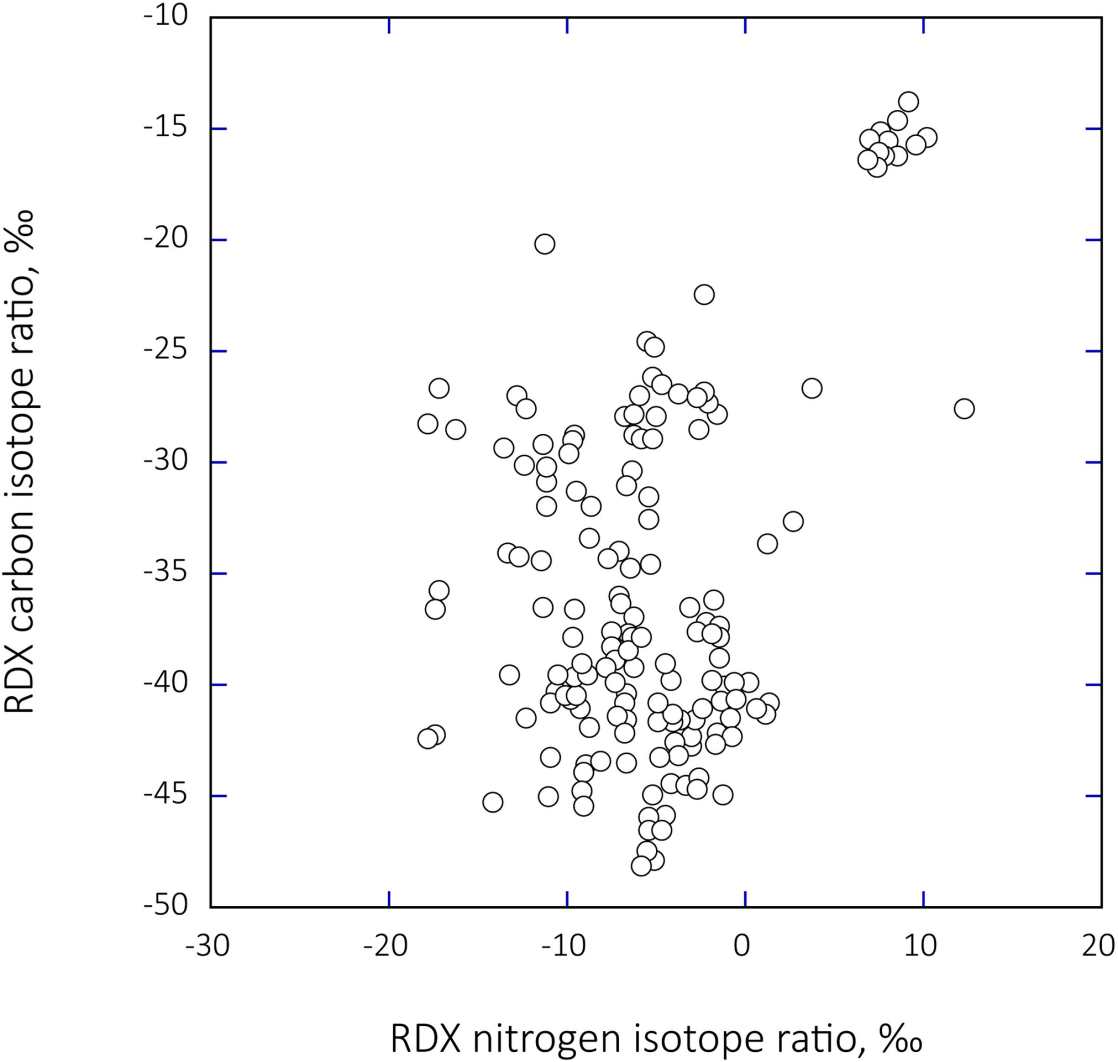
The δ^13^C and δ^15^N values of RDX extracted from different RDX‐containing explosives of known origin. Within this dataset, there are occasionally multiple acquisitions from a single manufacturer representing multiple manufacturing periods appearing as clusters. The RDX explosives were provided to us by the US Government and visits to several manufacturers. Source information remains the property of the US Government.

If stable isotope ratio analyses are to become useful as a forensic tool in analyses of postdetonation residues, there must be known variations among the δ^13^C and δ^15^N values of explosive materials. Stable isotope data from existing literature reveal extensive variations among TNT [8, 9, 10] and PETN [12] from different countries and manufacturing facilities. While Howa et al. [13, 32] provide data on chemical synthesis relationships leading to stable isotope variations in RDX, Figure 1 greatly expands the previously published variations in paired δ^13^C–δ^15^N values based on samples obtained from federal agencies and acquisitions from domestic and foreign production facilities. Both δ^13^C and δ^15^N values span approximately 35‰. Howa et al. [13, 32] explained the basis of these variations, which largely reflect differences in the sources of hexamine (δ^13^C) and differences in the termination point of the nitration reaction as well as the extent to which nitric acid is recycled between HMX/RDX batch syntheses (δ^15^N). Thus, if δ^13^C and δ^15^N values can be obtained from explosion residues and there are preblast and postblast δ^13^C/δ^15^N relationships, then stable isotope explosive residue analyses may be able to assist in forensic investigations.

### 3.2 Barrel Detonations

A set of experiments involving small detonations was conducted with different commercially available explosives as described above. Following detonations, sufficient explosive materials were recovered that allowed quantification and stable isotope analyses from individual swabs. In the three tables that follow, we provide δ^13^C and δ^15^N values for (a) undetonated (pre‐detonation) δ^13^C and δ^15^N values of the explosive compound; (b) postdetonation δ^13^C and δ^15^N values of the recovered explosive compound; and (c) the δ^13^C and δ^15^N differences between individual swab observations and that of the undetonated explosive. These data respond to Questions 1 and 2 from the introduction.

**FIGURE 2:**
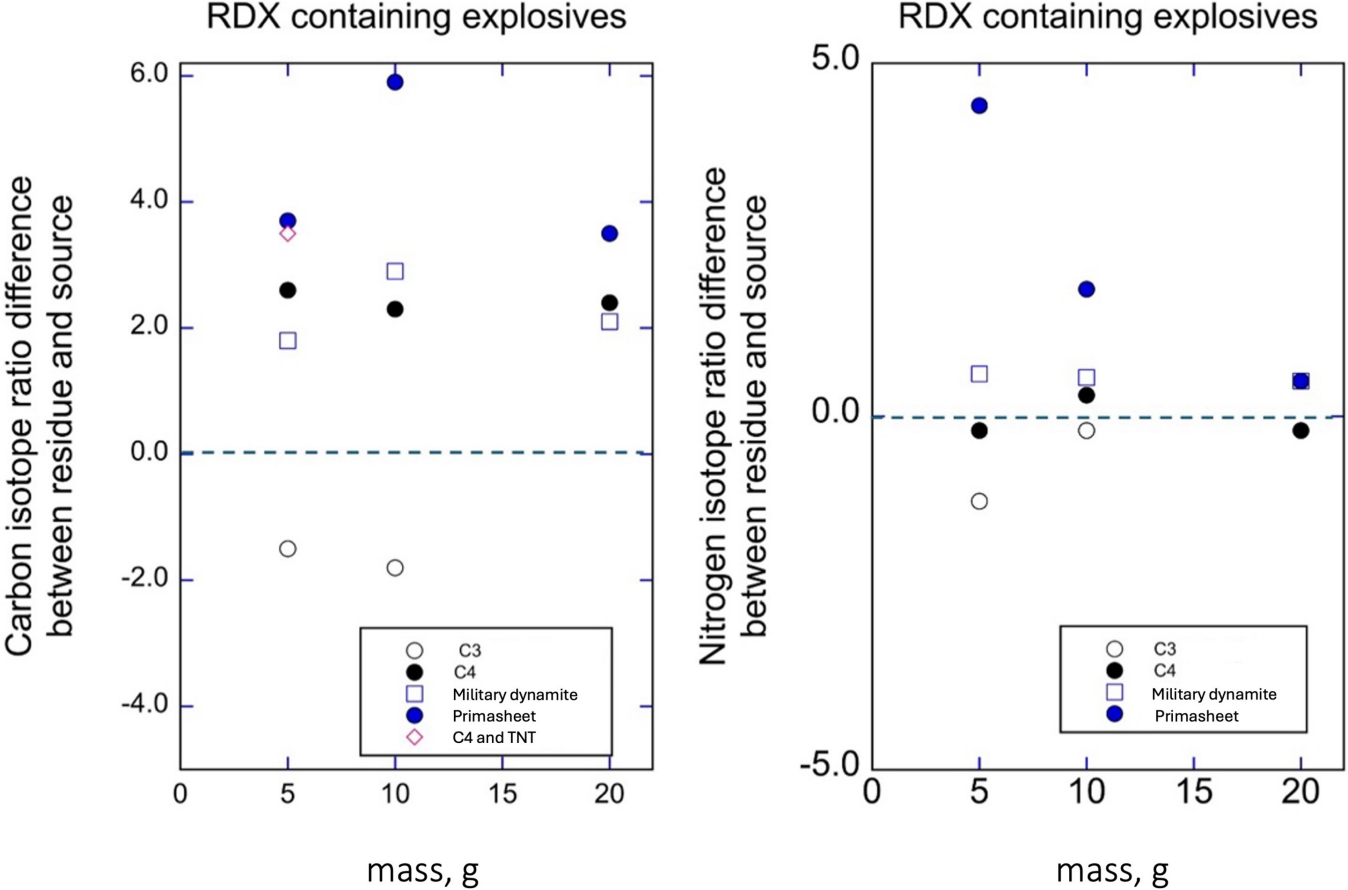
(Left) The differences in δ^13^C values between RDX in residues versus preblast RDX values as a function of the RDX mass (g). (Right) The differences in δ^15^N values between RDX in residues versus preblast RDX values as a function of the RDX mass (g).

Table 1 presents the data acquired when the detonated explosive was RDX. In 13 of the 15 experiments, individual swabs provided sufficient material (50+ μg) for stable isotope ratio determinations. These data are plotted in Figure 2 as the average δ^13^C and δ^15^N differences between detonated and undetonated RDX values. In 8 of the detonations, RDX δ^13^C values recovered from residues were statistically ^13^C enriched relative to the undetonated explosive. The C3 explosive was the exception. Those samples were 50–60 years old and were synthesized using a different process from the Bachmann process that is in common use today [32]. Considering the RDX samples produced using production pathways in use today, they averaged a 1.96‰ δ^13^C enrichment above their source values. In contrast, the RDX δ^15^N values recovered from residues were all similar to that of undetonated RDX values. Two of the 11 detonations were significantly different from source values. In contrast, in nine of the 11 experiments with RDX, δ^15^N values of residues were not significantly different from their source values.

**TABLE 1:** The results of explosive experiments where the primary explosive material was RDX. The experiment name refers to the explosive material used with the last numbers indicating the grams of the explosive in the experiment. Sampled and detected *n* refer to the number of swabs collected from barrel(s) and the number of swab samples containing explosive material. Each barrel had six sets of swabs. “r.n.d.” indicates that none of the swabs contained explosive material. Data are means and standard deviations. Asterisks indicate that the residue stable isotope ratio value is significantly different from zero (*t* test, *p* < 0.05), whereas nonsignificant different are denoted with NS.

Experiment name	Explosive	Sampled *n*	Detected *n*	Source carbon isotope ratio, ‰	Source nitrogen isotope ratio, ‰	Residue carbon isotope ratio, ‰	Residue nitrogen isotope ratio, ‰	Difference in carbon isotope ratio values, ‰	Difference in nitrogen isotope ratio values, ‰
C3 5	RDX	6	5	−15.3 ± 0.1	7.9 ± 0.2	−16.8 ± 1.0	6.7 ± 0.5	−1.5 ± 1.0^NS^	−1.2 ± 0.5*
C3 10	RDX	6	4	−15.3 ± 0.1	7.9 ± 0.2	−17.1 ± 0.5	7.7 ± 0.2	−1.8 ± 0.5*	−0.2 ± 0.2^NS^
C3 20	RDX	6	0	−15.3 ± 0.1	7.9 ± 0.2	r.n.d.	r.n.d.		
C4 5	RDX	24	20	−41.9 ± 0.6	−2.5 ± 0.1	−39.3 ± 0.8	−2.7 ± 0.6	2.6 ± 0.8*	−0.2 ± 0.6^NS^
C4 10	RDX	6	3	−41.9 ± 0.6	−2.5 ± 0.1	−39.6 ± 0.5	−2.8 ± 0.3	2.3 ± 0.5*	0.3 ± 0.3^NS^
C4 20	RDX	6	2	−41.9 ± 0.6	−2.5 ± 0.1	−39.7 ± 1.1	−2.7 ± 0.1	2.3 ± 1.1*	−0.2 ± 0.1 ^NS^
Military dynamite 5	RDX	6	5	−37.4 ± 0.1	−2.0 ± 0.1	−35.6 ± 0.5	−1.4 ± 0.2	1.8 ± 0.5*	0.6 ± 0.2*
Military dynamite 10	RDX	6	5	−37.4 ± 0.1	−2.0 ± 0.1	−34.5 ± 1.4	−1.6 ± 0.5	2.9 ± 1.4*	0.5 ± 1.7 ^NS^
Military dynamite 20	RDX	6	3	−37.4 ± 0.1	−2.0 ± 0.1	−35.3 ± 0.2	−1.4 ± 0.5	2.1 ± 0.2*	0.6 ± 0.5 ^NS^
Primasheet RDX 5	RDX	6	1	−40.3 ± 0.7	−10.5 ± 1.0	−36.3	−9.8	3.7	4.4
Primasheet RDX 10	RDX	6	1	−40.3 ± 0.7	−10.5 ± 1.0	−34.4	−8.7	5.9	1.8
Primasheet RDX 20	RDX	6	2	−40.3 ± 0.7	−10.5 ± 1.0	−36.9 ± 0.9	−9.2 ± 1.1	3.5 ± 0.9*	0.5 ± 1.1 ^NS^
Semtex H 5	RDX	24	4	−36.9 ± 4.3	2.3 ± 0.3	−36.1 ± 2.1	2.0 ± 0.5	0.9 ± 1.0 ^NS^	−0.4 ± 0.5 ^NS^
	PETN	24	0	−44.6 ± 0.7	−23.3 ± 0.9	r.n.d.	r.n.d.		
Semtex H 10	RDX	6	0	−36.9 ± 4.3	2.3 ± 0.3	r.n.d.	r.n.d.		
	PETN	6	0	−44.6 ± 0.7	−23.3 ± 0.9	r.n.d.	r.n.d.		
TNT and C4 10	RDX	12	7	−41.9 ± 0.6	−2.5 ± 0.1	−38.4 ± 2.0	−2.8 ± 0.5	3.5 ± 2.0 ^NS^	−0.3 ± 0.5 ^NS^
	TNT	12	0	−25.6 ± 0.1	9.5 ± 0.2	r.n.d.	r.n.d.	—	

**FIGURE 3:**
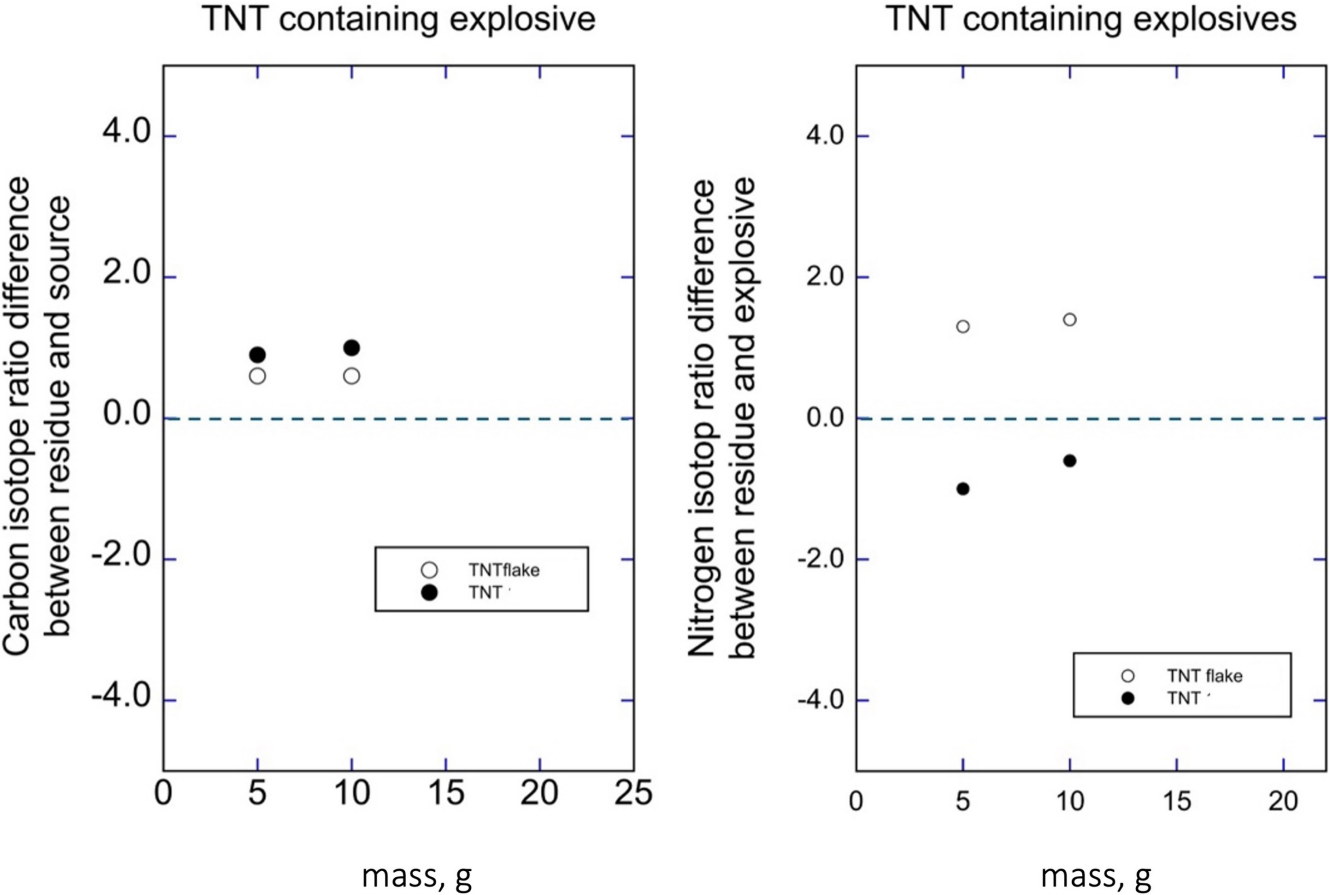
(Left) The differences in δ^13^C values between TNT in residues versus preblast TNT values as a function of the TNT mass (g). (Right) The differences in δ^15^N values between TNT in residues versus preblast TNT values as a function of the TNT mass (g).

Table 2 presents the data acquired when the detonated explosive was TNT. In seven of the nine experiments, individual swabs provided sufficient material (50+ μg) for stable isotope ratio determinations. These data are presented in Figure 3, where we plot the δ^13^C and δ^15^N differences between detonated and undetonated TNT. In only one of the four detonations were TNT δ^13^C values of residues ^13^C enriched relative to the undetonated explosive; otherwise, the δ^13^C values of TNT residues were indistinguishable from source materials. The TNT and flake TNT δ^15^N values recovered from residues were significantly different in two of the four detonations. With such a limited sample size, we cannot conclude that TNT δ^13^C and δ^15^N values differ in residues versus undetonated samples.

**TABLE 2:** The results of explosive experiments where the primary explosive material was TNT. The experiment name refers to the explosive material used with the last numbers indicating the grams of the explosive in the experiment. Sampled and detected *n* refer to the number of swabs collected from barrel(s) and the number of swab samples containing explosive material. Each barrel had six sets of swabs. “r.n.d” indicates that none of the swabs contained explosive material. Data are means and standard deviations. Asterisks indicate that the residue stable isotope ratio value is significantly different from zero (*t* test, *p* < 0.05), whereas nonsignificant different are denoted with NS.

Experiment name	Explosive	Sampled *n*	Detected *n*	Source carbon isotope ratio, ‰	Source nitrogen isotope ratio, ‰	Residue carbon isotope ratio, ‰	Residue nitrogen isotope ratio, ‰	Difference in carbon isotope ratio values, ‰	Difference in nitrogen isotope ratio values, ‰
TNT 5	TNT	12	12	−27.5 ± 0.0	8.3 ± 0.4	−26.6 ± 0.6	7.3 ± 0.7	0.9 ± 0.6 ^NS^	−1.0 ± 0.7 ^NS^
TNT 10	TNT	12	12	−27.5 ± 0.0	8.3 ± 0.4	−26.5 ± 0.4	7.7 ± 0.6	1.0 ± 0.4*	−0.6 ± 0.6 ^NS^
Military TNT 35	TNT	12	1	−27.5 ± 0.0	8.3 ± 0.4	−24.6	1.0	2.9	−7.3
TNT flake 5	TNT	6	6	−25.6 ± 0.1	9.5 ± 0.2	−25.2 ± 0.3	10.9 ± 0.2	0.5 ± 0.3 ^NS^	1.4 ± 0.2*
TNT flake 10	TNT	6	3	−25.6 ± 0.1	9.5 ± 0.2	−25.0 ± 0.4	10.9 ± 0.2	0.6 ± 1.4 ^NS^	1.4 ± 0.2*
TNT flake 20	TNT	6	0	−25.6 ± 0.1	9.5 ± 0.2	r.n.d.	r.n.d.		

Table 3 provides the results of detonation experiments where the primary explosive material was PETN. We were unable to detect PETN in any of the residues sampled.

**TABLE 3:** The results of explosive experiments where the primary explosive material was PETN. The experiment name refers to the explosive material used with the last numbers indicating the grams of the explosive in the experiment. Sampled and detected *n* refer to the number of swabs collected from barrel(s) and the number of swab samples containing explosive material. Each barrel had six sets of swabs. “r.n.d” indicates that none of the swabs contained explosive material. Data are means and standard deviations.

Experiment name	Explosive	Sampled *n*	Detected *n*	Source carbon isotope ratio, ‰	Source nitrogen isotope ratio, ‰	Residue carbon isotope ratio, ‰	Residue nitrogen isotope ratio, ‰
Det cord 5	PETN	6	0	−43.0 ± 0.1	−11.3 ± 1.1	r.n.d.	r.n.d.
Det cord 10	PETN	6	0	−43.0 ± 0.1	−11.3 ± 1.1	r.n.d.	r.n.d.
Det cord 20	PETN	6	0	−43.0 ± 0.1	−11.3 ± 1.1	r.n.d.	r.n.d.
Flex‐X M118 5	PETN	6	0	−39.3 ± 0.2	2.7 ± 1.0	r.n.d.	r.n.d.
Flex‐X M118 10	PETN	6	0	−39.3 ± 0.2	2.7 ± 1.0	r.n.d.	r.n.d.
Flex‐X M118 20	PETN	6	0	−39.3 ± 0.2	2.7 ± 1.0	r.n.d.	r.n.d.
Flex‐X 186 5	PETN	6	0	−38.3 ± 2.4	−7.0 ± 1.4	r.n.d.	r.n.d.
Flex‐X 186 10	PETN	6	0	−38.3 ± 2.4	−7.0 ± 1.4	r.n.d.	r.n.d.
Flex‐X 186 20	PETN	6	0	−38.3 ± 2.4	−7.0 ± 1.4	r.n.d.	r.n.d.
Primasheet PETN 5	PETN	6	0	−43.7 ± 0.8	−10.1 ± 0.6	r.n.d.	r.n.d.
Primasheet PETN 10	PETN	6	0	−43.7 ± 0.8	−10.1 ± 0.6	r.n.d.	r.n.d.
Primasheet PETN 20	PETN	6	1	−43.7 ± 0.8	−10.1 ± 0.6	r.n.d.	r.n.d.
Semtex 10 5	PETN	6	0	−44.1 ± 0.1	−24.4 ± 0.1	r.n.d.	r.n.d.
Semtex 10 20	PETN	6	0	−44.1 ± 0.1	−24.4 ± 0.1	r.n.d.	r.n.d.
Semtex A #13 5	PETN	6	0	−43.8 ± 0.2	−23.7 ± 0.3	r.n.d.	r.n.d.
Semtex A #13 20	PETN	6	0	−43.8 ± 0.2	−23.7 ± 0.3	r.n.d.	r.n.d.
Semtex A #26 5	PETN	6	0	−44.3 ± 0.1	−25.8 ± 0.2	r.n.d.	r.n.d.
Semtex A #26 20	PETN	6	0	−44.3 ± 0.1	−25.8 ± 0.2	r.n.d.	r.n.d.

In trying to explain why residue RDX δ^13^C values were enriched relative to undetonated material (Figures 2), we considered two possibilities. First, if the explosive material remained in solid form as it was projected away during the detonation, then perhaps the soon‐to‐be residue was undergoing sublimation as the explosion progressed. Second, if the detonating material was in a liquid form, then perhaps the soon‐to‐be residue was in a liquid phase undergoing vaporization as it was being blasted away. Both possibilities predict that the preferential loss of both ^12^C and ^14^N containing explosive molecules would vaporize from the soon‐to‐be residue material faster than in molecules dominated by ^13^C and ^15^N, leaving an explosive residue that was both ^13^C and ^15^N enriched. However, while the residues of RDX were ^13^C enriched, there was no indication that the residues were ^15^N enriched with increasing mass of the detonated explosive, leaving us with no clear explanations for the observations in Figures 2. However, it is worth noting that McGuire et al. [28] observed a similar pattern, with δ^13^C values of RDX (a nonaromatic explosive) residues enriched relative to the source, whereas TNT (an aromatic‐based explosive) residue δ^13^C values were similar to those of the source materials.

**FIGURE 4:**
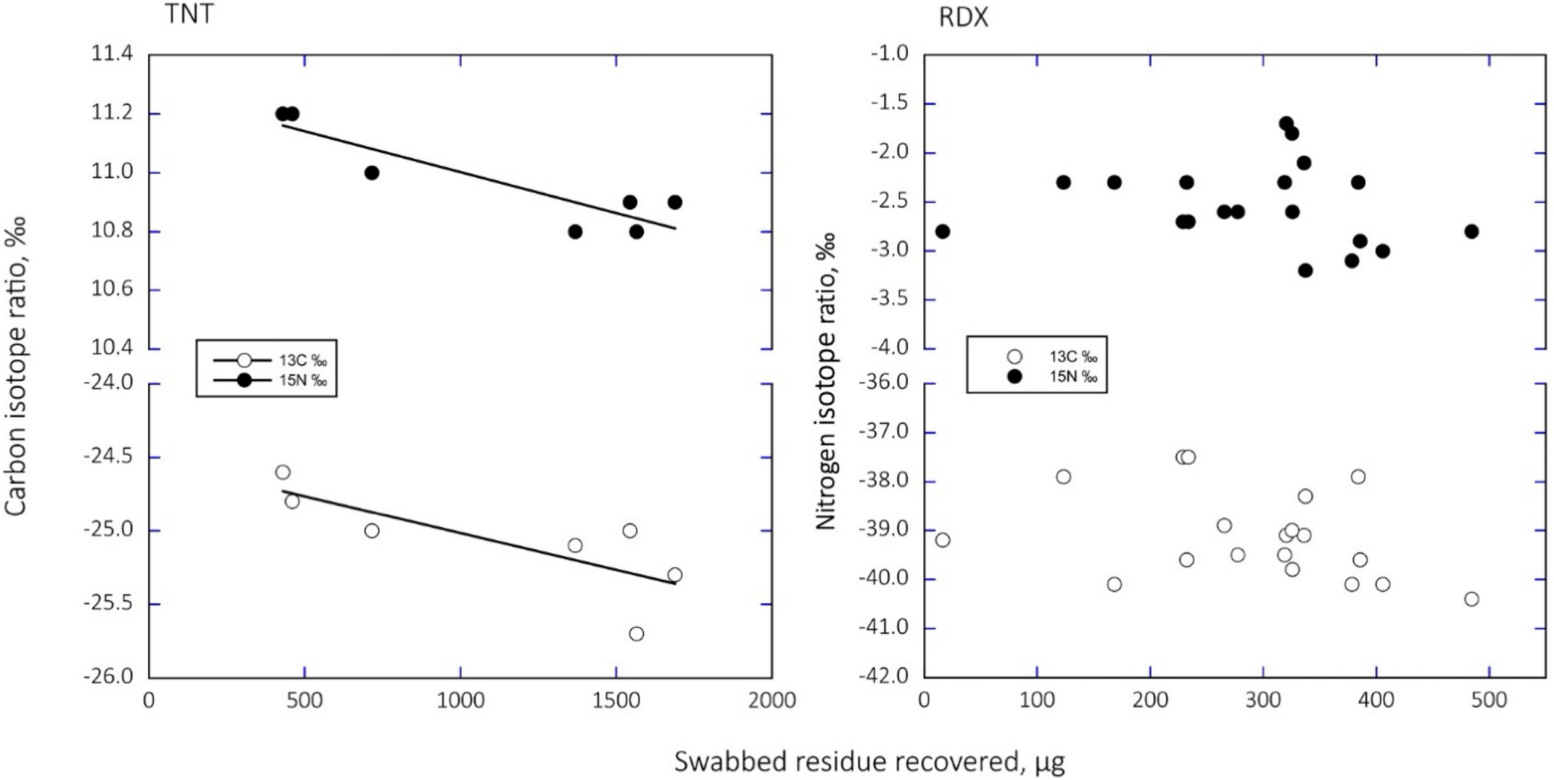
The δ^13^C and δ^15^N values of TNT recovered in a swab (left) and the δ^13^C and δ^15^N values of RDX recovered in a swab (right) as functions of the amount in the explosive recovered in the swab. The relationship for TNT recovered were statistically significant for both δ^13^C (*R* ^2^ = 0.53, *p* = 0.0037) or δ^15^N (*R* ^2^ = 0.77, *p* = 0.006). The relationship for RDX recovered were not statistically significant for either δ^13^C (*p* = 0.16) or δ^15^N (*p* = 0.57).

Figure 4 plots the observations between stable isotope ratios of TNT (left) and RDX (right) versus the amount of explosive material recovered from individual swabs. The relationships between δ^13^C and δ^15^N values of TNT exhibited statistically significant increases as the amounts of recovered residues declined. This observation is consistent with the possibility that recovered residues represent evidence of vaporized enrichment relative to the source explosive. However, the relationships between δ^13^C and δ^15^N values of RDX were both ^13^C and ^15^N enriched relative to the source explosive, but there was no statistically significant relationship with the amounts of residue recovered.

### 3.3 Mark‐84 Detonations

In a second set of field experiments, 2000‐pound Mark‐84 bombs were detonated at remote locations within a US Government facility. As a test of the feasibility that stable isotope ratio measurements would be useful in postblast analyses under field conditions, we were able to obtain postdetonation soil samples. Based on the barrel experiments, our expectations were that δ^13^C and δ^15^N values of any residues recovered from the soil would be similar or enriched relative to the source materials. Unfortunately, we did not have access to the pre‐detonation explosive materials. However, following each of the detonations we were provided with 1 kg of surface soils, collected within 3 days from the perimeters of each crater. Only 250 g of soil was required for HPLC and IRMS analyses. HMX, RDX, and NT were recovered from the perimeter soils.

Carbon and nitrogen isotope ratios of HMX residues across the five different craters were similar with the mean values of −42.7‰ and −2.6‰, respectively, with a small variance among detonations (Table 4). This result suggested that the stable isotope ratios of HMX from each of the bombs were likely to have been similar and perhaps identical to each other. Thus, there is a high probability that each of these bombs came from the same manufacturer during a similar production period and therefore might be expected to have similar isotope ratio compositions. This cross‐crater pattern was also seen in the carbon and nitrogen isotope ratio values of both RDX (δ^13^C = −41.3‰, δ^15^N = −1.8‰, mean values) and combined nitrotoluene compounds (δ^13^C = −24.3‰, δ^15^N = +7.8‰, mean values) (Table 4).

**TABLE 4:** The δ^13^C and δ^15^N values of HMX, RDX, and NT recovered from residues collected from crater rims following detonations of Mark‐84 bombs. NT is an amalgamation of TNT, 2‐amino‐4,6‐, and 2,4‐dinitrotoluene. Data are means ± 1 standard deviation (*n* = 3).

	HMX	HMX	RDX	RDX	NT	NT
Crater	δ^13^C, ‰	δ^15^N, ‰	δ^13^C, ‰	δ^15^N, ‰	δ^13^C, ‰	δ^15^N, ‰
A	−42.8 ± 0.6	−2.7 ± 0.3	−39.9 ± 0.3	−2.2 ± 0.4	−23.7 ± 0.1	8.3 ± 0.5
B	−43.3 ± 0.2	−27. ± 0.1	−41.8 ± 0.2	−1.6 ± 0.1	−23.5	9.1
C	−42.0 ± 4.3	−2.5 ± 0.3	−40.9 ± 0.4	−1.7 ± 0.6	−24.7 ± 0.1	7.1 ± 0.9
D	−42.7 ± 0.4	−2.7 ± 0.5	−42.5 ± 0.4	−2.1 ± 0.5	—	—
E	−42.7 ± 0.6	−2.3 ± 0.4	−41.0 ± 0.3	−1.3 ± 0.3	−25.4 ± 0.2	6.8 ± 1.4

**FIGURE 5:**
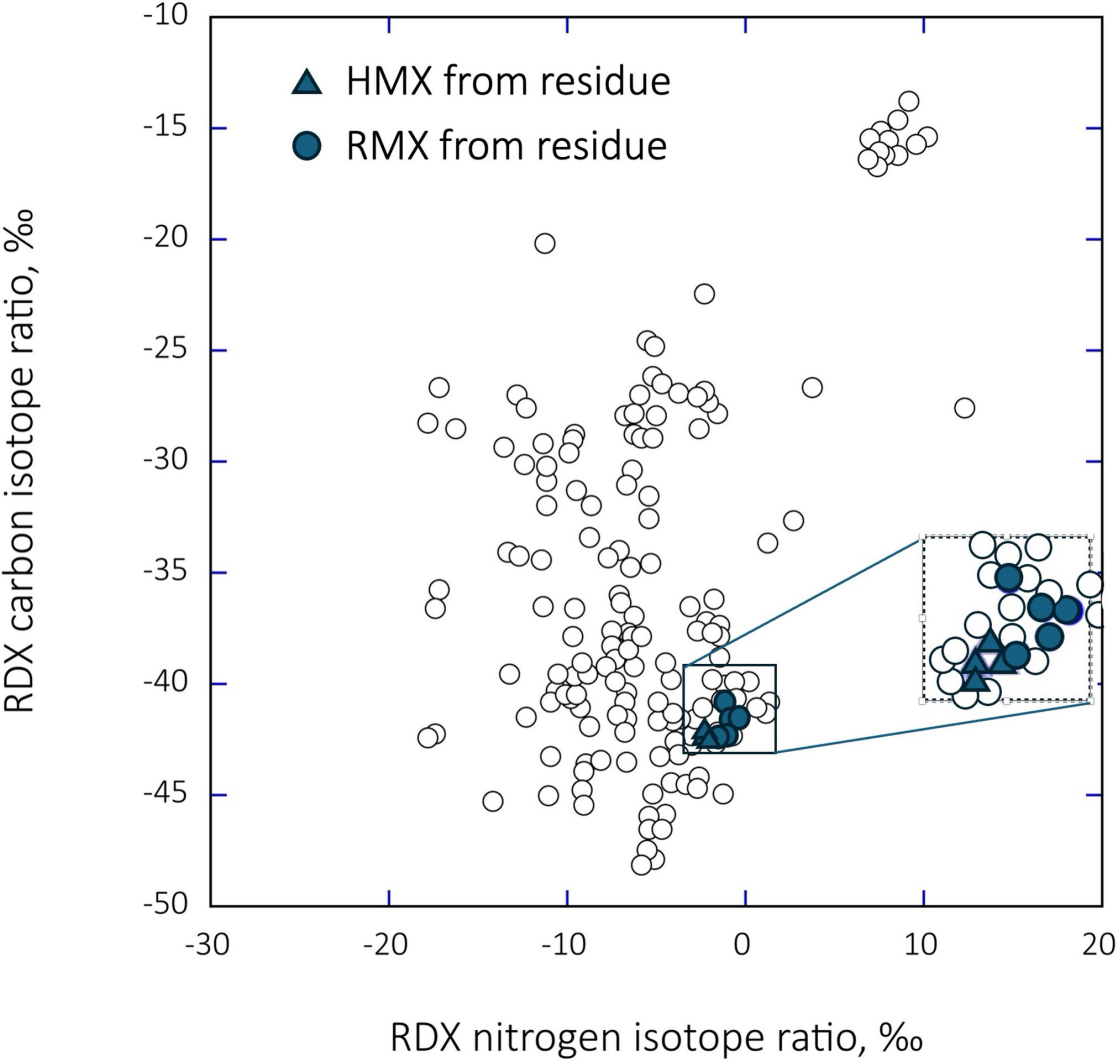
The δ^13^C and δ^15^N values of HMX and RDX residues extracted from soil perimeters following Mark‐84 detonations (in blue circles and triangles) with data from Figure 1 (unfilled circles). Each of the blue circles (RDX) and blue triangles (HMX) represents the results for a single detonation. The unfilled circles within the boxes represent the stable isotope ratio observations for RDX/HMX samples that were acquired from different production runs at the Holston Army Ammunition Plant.

When the paired carbon and nitrogen isotope ratios of explosive residues recovered from each of the craters are plotted, it becomes clear that the data for the three explosive types fall into cluster groups (Figure 5). The proximity of the HMX and RDX carbon‐nitrogen isotope ratio values across groupings would be expected if the HMX and RDX had been produced by the Bachmann process [32].

**FIGURE 6:**
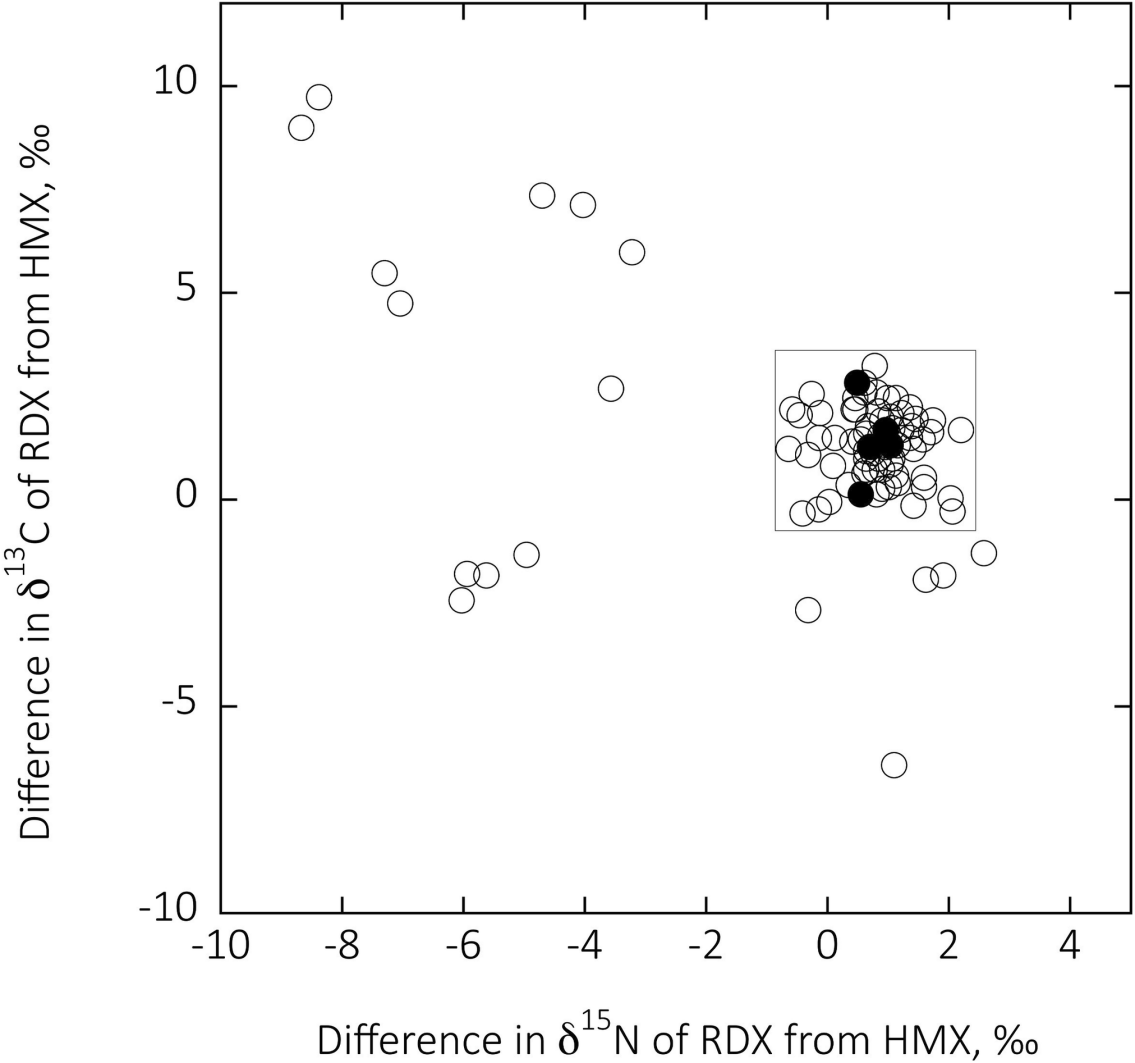
Open circles represent differences in δ^13^C values between HMX and RDX in an explosive containing both HMX and RDX plotted as a function of the difference in δ^15^N values between HMX and RDX. The data presented represent all known observations of authentic acquisitions from the database shown in Figure 1. Solid circles represent soil residue observations. Those observations encompassed within the area described by the square are HMX/RDX explosives synthesized during the same production process.

We have observed similar RDX‐HMX isotope patterns before from explosive manufacturers that produce RDX and HMX during the same production cycle, as opposed to factories that separately manufacture RDX and HMX and subsequently mix the two together. If RDX and HMX are produced during separate production periods, then there is typically a greater difference in the carbon‐nitrogen isotope pairings of RDX and HMX. In Figure 6, we have plotted the difference in the carbon isotope ratios between RDX and HMX (RDX value minus HMX value) from different explosives in the database presented in Figure 1 as a function of the difference in the nitrogen isotope ratios between RDX and HMX (RDX value minus HMX value) in that same explosive. For comparison, the data in Table 4 also show small stable isotope ratio differences measured from the different soil samples. The tight cluster of isotopic observations within our soil crater samples suggests that both explosive compounds were manufactured simultaneously at the same production facility. Each of the apparent outliers in Figure 6 represents RDX/HMX explosive mixtures in which these two explosive compounds are synthesized separately and subsequently mixed together to form the final product.

These promising results suggest that it may be possible to combine composition information from explosive residue analyses and stable isotope ratios to provide insights into the chemical composition and manufacturer of explosives based on soil analyses following detonations. At this point, we cannot determine whether the stable isotope ratio of undetonated Mark‐84 bombs and recovered explosive residues would be expected to be identical; we can extrapolate from the barrel experiments (Tables 1 and 2). The degree of separation in preblast and postblast δ^13^C explosive/residue values may be different between aromatic‐based and nonaromatic explosives, as was observed earlier. For simplicity's sake, let us assume that there is a 1:1 relationship between undetonated materials and recovered explosive residues. If so, we can compare the stable isotope ratios of the explosives extracted from the soil at the craters with observations in the database (Figure 1).

There are 81 observations within Figure 1 that contain an explosive consisting of both RDX and HMX. If we use the mean isotope ratio values of RDX and HMX recovered from soils, we find the closest agreement between the soil samples and isotope ratio values in the database presented in Figure 1 is with the explosive Holston B4. The isotope agreement here for RDX and HMX isotope values is reasonable but offset in that residue values are consistently isotopically heavier. Both HMX and RDX are manufactured on site at Holston using the Bachman process. While the isotope values seem to be different for TNT between Holston B4 and soil samples, it is useful to note that TNT is usually purchased offsite by Holston from several different manufacturers (based on conversations at Holston several years ago). It is further useful to note that the soil residue nitrotoluene values were again heavier than those observed in the Holston B4 samples. Together these data suggest that the residue isotope ratio values may be systematically heavier than expected for the undetonated materials and consistent with the barrel observations for nonaromatic explosives (HMX, RDX) but not for nonaromatic explosives (TNT).

The consistency of stable isotope ratio values between Holston B4 and soil samples suggests the potential that Question 3 in the introduction is approachable. However, it is also possible that the database in Figure 1 does not adequately represent a sufficient number of manufacturers and that there could be overlap in δ^13^C and δ^15^N RDX values among different manufacturers. At this point, the results of our field experiments suggest that there is a small ^13^C enrichment in HMX and RDX δ^13^C values during detonation, but no ^15^N enrichment in residue δ^15^N values. Thus, while speculative, the soil and database observations are consistent with the suggestion that these Mark‐84 bombs included HMX/RDX explosives manufactured at the Holston Facility.

## Author Contributions

James R. Ehleringer designed and conducted the field experiments. John D. Howa and Mr. Michael Lott conducted the analyses. James R. Ehleringer and John D. Howa wrote the manuscript.

## Funding

This work was supported by the US Department of Defense (N41756‐00C‐0901 and 2002‐H151200‐000).

## Data Availability

Data used in presenting this manuscript are available upon written request.
